# Low frequency ultrasonic debridement : a new tool in our armoury?

**DOI:** 10.1186/1757-1146-4-S1-P7

**Published:** 2011-05-20

**Authors:** Gillian Butcher

**Affiliations:** 1 Acute Podiatry Services Manager, Southern Health, Victoria, 3168, Australia

## 

Low Frequency Ultrasound Debridement (LFUD) provided by the Sonoca 185 is a new method of debriding wounds that is less traumatic, less painful, and can achieve faster healing rates (mean 2.5 times faster in prospective randomised trials vs. theatre sharps). It is bactericidal and enables many debridement cases to be performed by a podiatrist or wound consultant in a less resource intensive setting (e.g. bed-side or outpatient clinic) than by a surgeon in theatre. Essentially the Sonoca 185 is a “high technology knife” that has numerous advantages: safety, efficacy, resource utilisation, cost savings, faster healing and new capabilities over existing practice. The vibrating handpiece creates an ultrasound wave delivered to the wound in a saline medium that in turn creates two effects close to the wound surface: (i) acoustic streaming (fluid motion at the boundary of the effect) and (ii) cavitation (imploding “gaps” in the fluid that create micro-shockwaves). In turn the two effects of *cavitation* and *acoustic streaming* result in three clinical effects (i) debridement, (ii) wound healing stimulatory effects, (iii) bactericidal effects.It is these three clinical effects of (i) atraumatic selective tissue debridement, (ii) wound stimulatory effects and (iii) antibacterial activity that create the clinical results. LFUD therapy using the Sonoca 185 has been implemented at Monash Medical Centre by the Podiatry Department and Wound Clinical Nurse Consultant as part of a 4-site Department of Health trial across Victoria. This presentation is intended to familiarise you with this treatment modality by presenting a brief literature review and providing case presentations to show the benefits of adding this tool to your wound care armoury. It will also provide a brief overview of the trial, how it was funded under the Victorian Policy Advisory Committee on Clinical Practice and Technology (VPACT) and the supports that you as clinicians must have in place should you wish to progress down this path as a funding option for future services.

